# Radiographic and Histomorphologic Evaluation of the Maxillary Bone after Crestal Mini Sinus Lift Using Absorbable Collagen—Retrospective Evaluation

**DOI:** 10.3390/dj10040058

**Published:** 2022-04-02

**Authors:** Saverio Cosola, Biagio Di Dino, Tonino Traini, Young-Sam Kim, Young-Min Park, Simone Marconcini, Ugo Covani, Raffaele Vinci

**Affiliations:** 1 Department of Stomatology, Tuscan Stomatologic Institute, Foundation for Dental Clinic, Research and Continuing Education, 55041 Camaiore, Italy; s.cosola@hotmail.it (S.C.); simosurg@gmail.com (S.M.); covani@covani.it (U.C.); 2 Department of Medical Oral and Biotechnological Sciences, University ‘G. d’Annunzio’ of Chieti-Pescara, 66100 Chieti, Italy; tonino.traini@unich.it; 3 Gangam Dental Office, Seoul 06614, Korea; doctorkimys@gmail.com (Y.-S.K.); min1810@hotmail.com (Y.-M.P.); 4 Department of Dentistry, IRCCS San Raffaele, 20132 Milan, Italy; vinci.raffaele@hsr.it; 5 Post-Garduate School of Oral Surgery, Vita-Salute San Raffaele University, 20132 Milan, Italy

**Keywords:** bone histology, mini sinus lift, CBCT, radiographic evaluation, collagen, hyaluronic acid

## Abstract

Background: After tooth extraction, the alveolar bone loses volume in height and width over time, meaning that reconstructive procedures may be necessary to perform implant placement. In the maxilla, to increase the bone volume, a mini-invasive surgery, such as a sinus lift using the crestal approach, could be performed. Methods: A crestal approach was used in this study to perform the sinus lift, fracturing the bone and inserting collagen (Condress^®^). The single dental implant was placed in the healed bone after six months. Results: The newly formed bone was histologically analyzed after healing. Histomorphological analyses confirmed the quality of the new bone formation even without graft biomaterials. This is probably due to the enlargement of the space, meaning more vascularization and stabilization of the coagulum. Conclusion: Using just collagen could be sufficient to induce proper new bone formation in particular clinical situations, with a minimally invasive surgery to perform a sinus lift.

## 1. Introduction

After tooth extraction, the alveolar bone may resorb and lose volume in height and width due to the absence of function and the reduction of blood supply [1]. One year after dental avulsion, there is a loss of 50% of the width of the alveolar bone in the buccal-palatal/lingual directions, of which 30% is lost in the first three months, especially in the vestibular component. This resorption is due to the vestibular bone quality, which has a major cortical component; therefore, there is less vascularization and, subsequently, the alveolar ridge is also reduced in height [2]. In the phases of extraction, it is possible to perform surgery to reduce the physiological bone loss using various principles of ‘socket preservation’, such as atraumatic extraction and minimally invasive surgery, or the use of membranes to leave enough bone to perform an implant placement [3]. The resorption of the alveolar bone stabilizes between the first and second years from the extraction. However, although the percentage of bone loss in subsequent years is lower, the process continues throughout life [4]. If the alveolar socket is managed correctly, there is less bone resorption, especially in the first years. Nevertheless, without function, the edentulous sites may lose width, height, and density, resulting in reconstructive or regenerative surgery to replace prosthetic rehabilitation structures.

In the maxilla, there is not only a resorption of the alveolar bone, but also a physiological enlargement of the maxillary sinus due to age “pneumatization”. This lack of bone may be solved in modern implantology by different clinical approaches that can be used to perform a prosthetic rehabilitation for masticatory and phonetic purposes. Clinical studies and the last review of Ravidà and co-workers (2019) stated that short implants could be a valid option to rehabilitate an atrophic maxillary bone with a mean predictable success following up to a 3-year follow-up [5,6]. Other clinical options to treat maxillary atrophy include the reconstructive procedures involving, or not, biomaterials with lateral or crestal approaches [7,8].

In patients with medium and mild bone atrophy in the posterior regions of the maxillary bone, less invasive and more conservative surgical procedures are often preferred. Sinus floor elevation techniques with a crestal approach are used to achieve this result, both in association with graft biomaterials and without [9]. The main difference between the lateral window technique and the crestal approach is that the sinus membrane is lifted through the crestal bone using osteotomes, or it directly implants after the implant site’s preparation [10]. The crestal approach is classically called the osteotome-mediated transcrestal (SFE) technique, and it was proposed by Tatum in the 1970s. In 1986, Tatum modified this technique using site preparation to perform implant placement and then used the osteotome to elevate the sinus using a ‘greenstick fracture’, moving the implant in an apical direction [11]. Then, in 1994, Summers added to this technique the use of a unique set of osteotomes of varying diameters to increase the bone volume and perform the implant placement without using drills for the site preparation [12]. A recent histomorphometric study has reported on the healing phases of bone after the elevation of the sinus floor in an animal model [13].

In this study, a crestal approach was used to perform the sinus lift and fracture the bone, putting in the post-extractive site a lyophilized non-denatured equine type-I collagen (Condress^®,^, Smith & Nephew S.r.l., Monza, Italy) and an amino acid-based bioactive gel for the soft tissue healing with the aim to reconstruct the bone, leaving enough space to perform an implant placement in the healed hard tissue. There are no clinical studies reporting in the literature the histomorphometric evaluation after a crestal sinus lift using just collagen. The aim is to perform a histological analysis of the new bone that has been formed after a crestal sinus lift to evaluate the quality of the new bone formed using cone-beam computed tomography (CBCT) after 6 months of healing.

## 2. Materials and Methods

A total of 10 patients were included in the present retrospective evaluation according to the Declaration of Helsinki of 2008, updated in 2013. Each patient agreed to participate in the study by filling in a written informed consent form for a larger in-course study. The patients enrolled underwent a single extraction with crestal sinus lift using just absorbable collagen followed by a delayed implant placement performed after 6 months at the Tuscan Stomatological Institute, Camaiore, Italy. All patients received normal procedures to rehabilitate a single edentulism from the same surgeon and pre-operative and post-operative cone-beam computed tomography (CBCT) analyses were performed to evaluate the augmented bone after the crestal sinus lift. Patients were evaluated also using histomorphologic analyses to test the quality of the new formed bone. The endoral radiographs were taken at implant placement (baseline) and in other follow-ups every 1 year by using the Rinn holders blocked in a personalized silicone bite and with a long-cone paralleling technique to standardize the axis and position [14,15].

### 2.1. Inclusion/Exclusion Criteria

All 10 cases were adults aged between 40 and 65 years old. Patients younger than 40 years old rarely have deficiencies in their maxillary bone; therefore, no patients below 40 were enrolled. None of the included patients had systemic diseases or smoking habits, and thus did not have risk factors for implant failure. Immediately before the surgery, all patients used a mouth rinse of chlorhexidine digluconate solution 0.2% for 1 min (Curasept S.p.A., Varese, Italy) and 1 compress of antibiotic (875 mg of amoxicillin with 125 mg of clavulanic acid); then, after the surgery, they continued the antibiotic treatment (1 compress every 12 h) for 5 days more. After each surgery, for one week, patients were asked to apply an oral amino acid-based gel with hyaluronic acid (Aminogam gel^®^ of Polifarma Benessere S.r.l, Rome, Italy) to make the healing process faster, reducing discomfort and swelling.

### 2.2. Clinical and Radiological Outcomes

The bone distance between the crest and the sinus was calculated using CBCT.

Pain and complications that eventually occurred after surgery were registered thanks to patients’ questionnaires regarding the nasal pain (strong, moderate, or tolerable) or other maxillary sinus symptoms or other severe complications.

### 2.3. Surgical Procedures

At least two hours before surgery, each patient underwent an administration of 1 g amoxicillin; then, they were given 1 g twice daily for the next postoperative 5 days. Local anesthesia was administered via an injection of mepivacaine 15 mg/mL with adrenaline at 1:100,000.

The maxillary edentulous site or the post-extractive site were approached with the aim of maintaining the full integrity of the socket and to limit both the buccal and palatal flaps. The crestal sinus lift was performed by using a minimally invasive surgical instrument mounted on an electromagnetic device (Magnetic Mallet, www.osseotouch.com, accessed on 8 June 2016, Turbigo, Milano, Italy). This device was used to place in the site the equine collagen (Condress^®^) and to fracture the apical bone, keeping the sinus membrane. In the case of tooth extraction, a periodontal millimetrated probe was generally used to verify the integrity of the sockets. 

The patients enrolled received a delayed implant surgery after the sinus lift and the 6-month healing. The drilling to prepare the bone for the implant placement was performed with trepan burs to take the sample for the histomorphological analysis using azure B–methylene blue staining.

### 2.4. Statistical Analysis

Descriptive statistics for patients’ characteristics and treatment outcomes were conducted using Microsoft Excel, Office 365 (Microsoft Corp. Redmond, WA., USA, 2020). Student’s *t*-test (*p* < 0.05) was used to analyze the differences between subgroups according to the follow-up, complication, the clinical intervention, and the type of coronectomy (intentional or nonintentional).

## 3. Results

Over a period of 6 months, 10 patients were included in this study. The mean age of the included patients was 46.9 ± 2.85 (44–52) years old. Gender was equally distributed over the cohort, with five females (50.0%) and five males (50.0%). During the follow-up period, which ranged between 5 and 7 months, with a mean value of 6.7 ± 0.67 months, no patient complained about nasal pain or other maxillary sinus symptoms in the upper jaw after the first 2 weeks. No patients reported severe complications.

### Case Presented with Histomorphologic Evaluation

**Figure 1:**
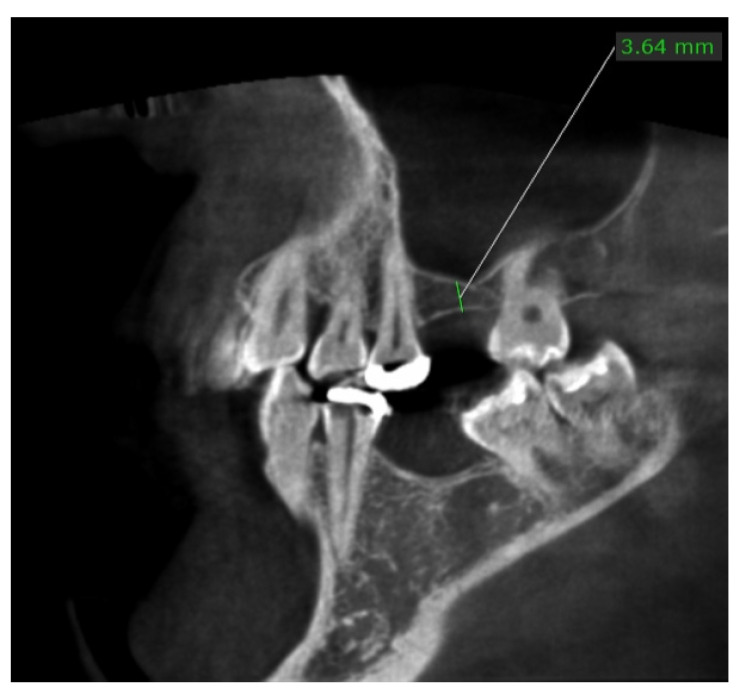
The section of the CBCT showing a residual height of 3.6 mm before the sinus lift at baseline.

The patient aged 45 years underwent a tooth extraction of the left first molar (element 26) due to a crown fracture. She had no systemic disease or other contraindication to rehabilitate the single edentulous site using a single implant–prosthetic rehabilitation, even though she did not accept the treatment plan for family and personal reasons. After two weeks, she asked for a prosthetic restoration of the same site, accepting the therapeutic plan for a fixed prosthesis on an osseointegrated implant. On baseline, clinicians performed surgical planning via 3D radiological examination. Cone-beam computed tomography (CBCT) showed a 3 mm residual bone height of bone available for implant placement (Figure 1).

On the same week, a sinus lift using the crestal approach was performed: after a crestal incision and a full flap between dental elements 25 and 27, a small drill was used to prepare the implant site (Drill-Kit, MICERIUM S.p.A., Genova, Italy), but instead of proceeding to the implant placement, only equine collagen was placed (Condress^®^). The site preparation was performed until the sinus membrane was visible. After the collagen insertion, two sheets of collagen sponge cut into strips of about 5 mm wide were placed gently in an apical direction, and the future implant site was sutured by two detached mattress stitches, and a gel with hyaluronic acid was applied (Aminogam gel^®^).

**Figure 2:**
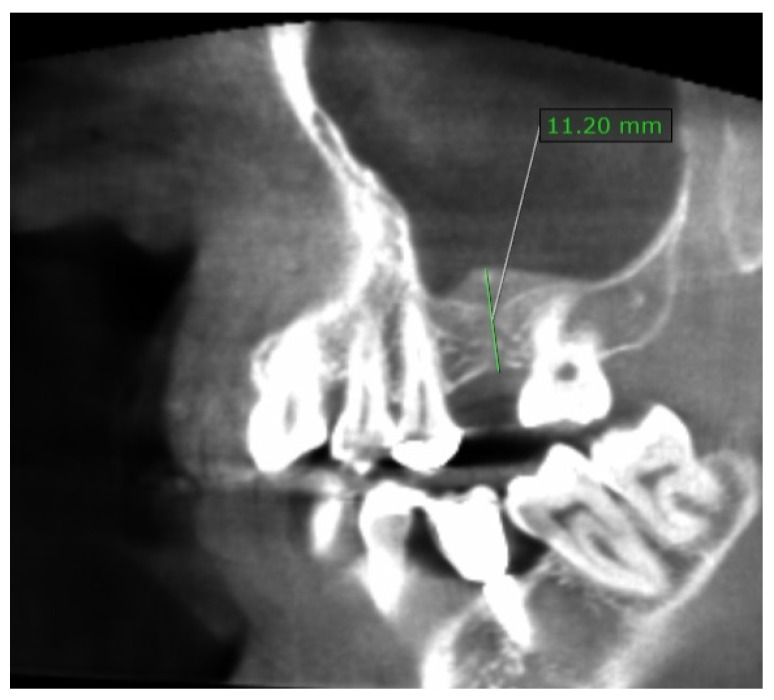
The section of the CBCT showing a residual height of 11.2 mm after 6 months from the sinus lift, meaning an earning bone height of 7.6 mm. The bone seems to be of good quality for dental implant placement.

After 6 months from baseline, CBCT was performed again to check the level of bone regeneration (Figure 2) and to plan implant placement in the 26 sites. The radiographically visible bone gain was approximately 6 mm.

**Figure 3:**
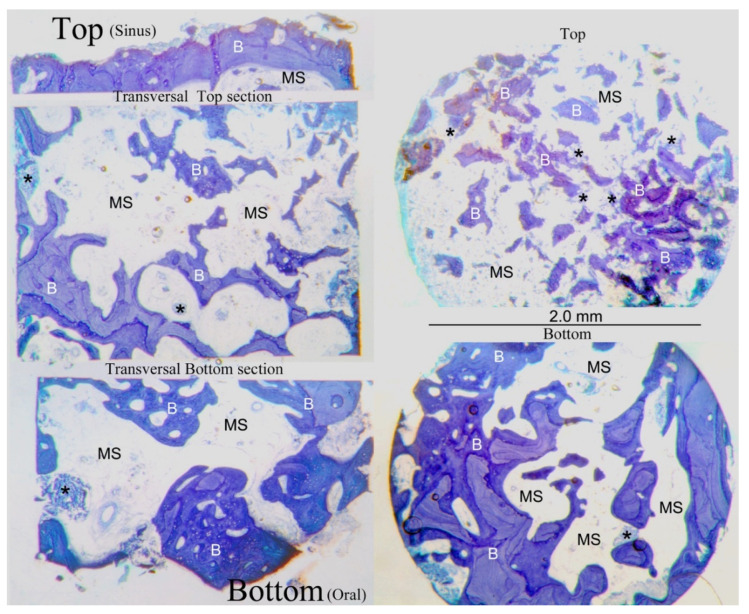
Low-power magnification (9×) of the bone core in the longitudinal section appeared to be well organized with a trabecular network (B) and several distributed marrow spaces (MS). No biomaterial remnants were reported. azure B–methylene blue staining was used.

In Figure 3, the bone core in the longitudinal section is generally organized with an excellent trabecular network (B) and several distributed marrow spaces (MS). No biomaterial remnants were visible because it was probably totally absorbed.

**Figure 4:**
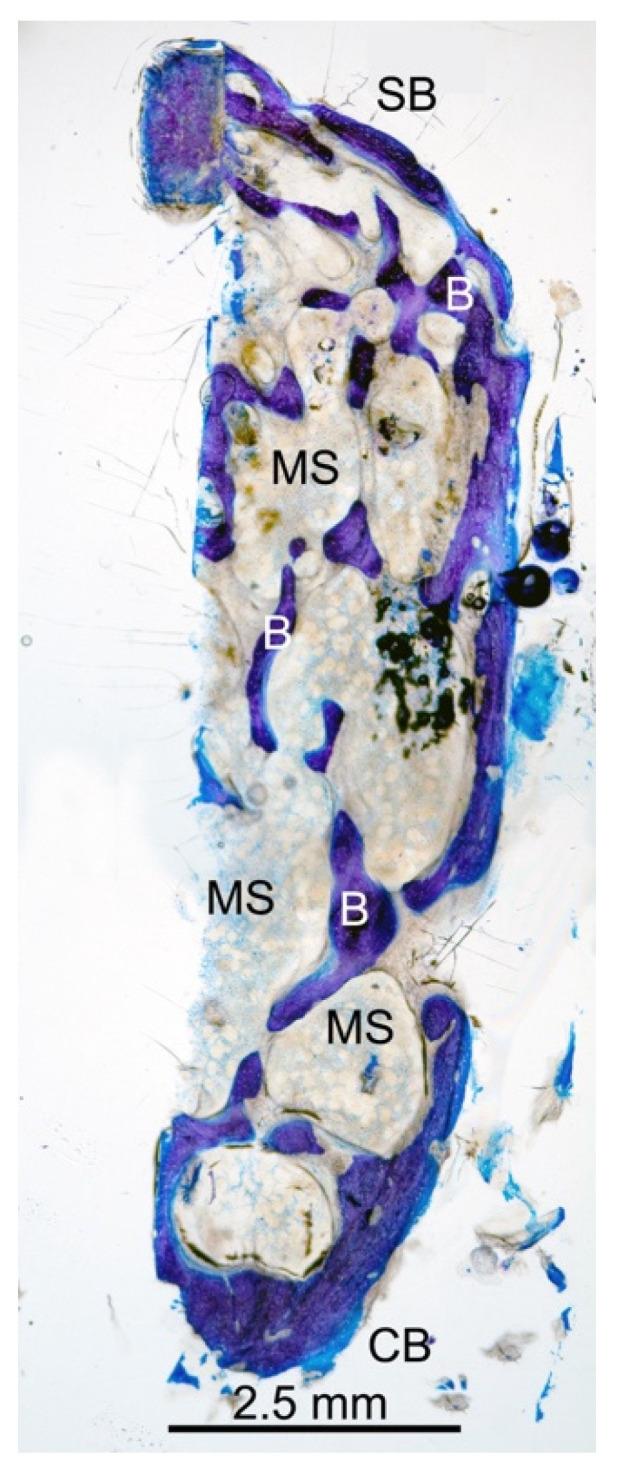
Low-power magnification (12×) of the longitudinal central section of the bone core. A fine trabecular bone network (B) and distributed marrow spaces (MS) were seen. Small remnants of biomaterial were present. (CB) crestal bone; (SB) sinus bone; azure B–methylene blue staining was used.

In Figure 4, the bone core in the longitudinal section appears to be well organized with a trabecular network (B) and several distributed marrow spaces (MS).

**Figure 5:**
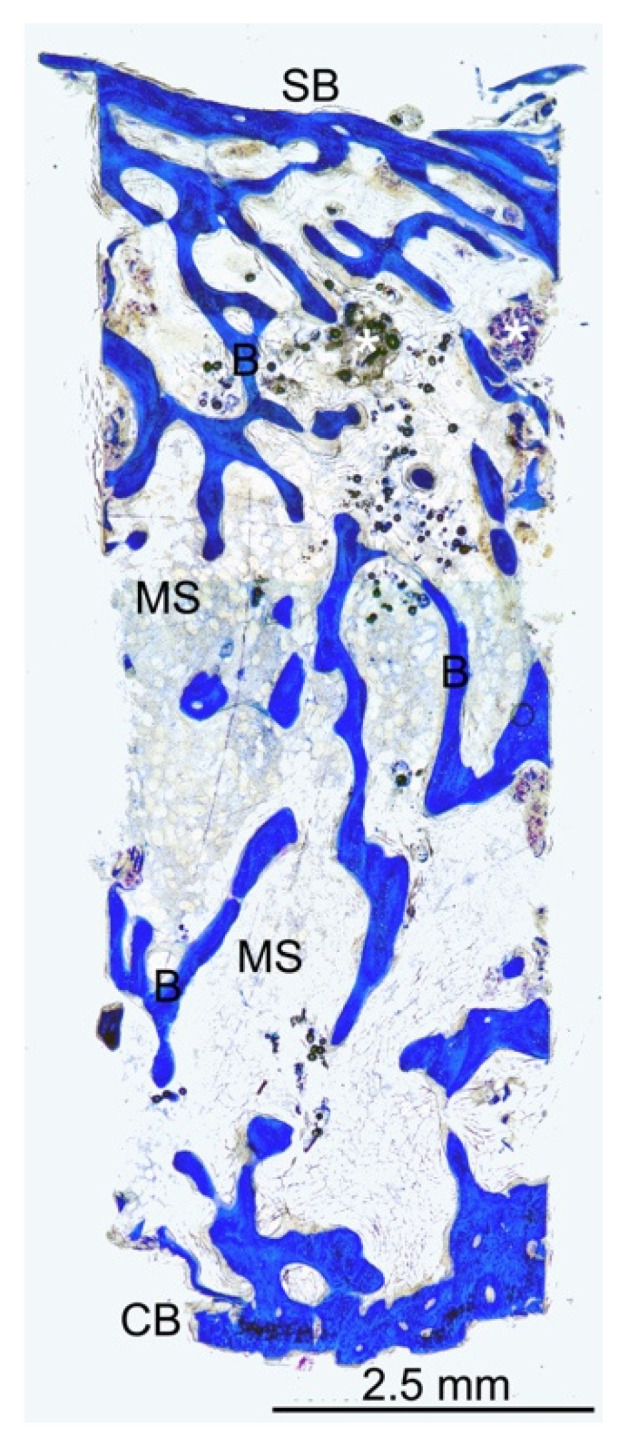
At low-power magnification (20×), the transversal and top and bottom sections were related to the longitudinal central section of the bone specimen to best analyze the bone microstructure of the regenerated area. The source sites of the transversal sections were visible and labelled, along with the longitudinal ones. The bone microstructure appeared to be well organized, with thicker bone trabeculae (B) that were mainly present in the bottom areas, while under the bone sinus floor, very thin and distributed bone trabeculae were highlighted. The marrow spaces (MS) were well distributed along all the sections. Several small remnants of biomaterial (*) were present mainly under the sinus floor bone. Azure B–methylene blue staining was used. (CB) crestal bone; (SB) sinus bone.

No biomaterial evidence is present in Figure 5 due to the collagen sponge being entirely resorbable, whereas the xenogenous biomaterials based on deproteinized bovine bone mineral that are applied for maxillary sinus elevation leave in place remnants after the bone healing phase. The bone microstructure appeared to be well organized with thicker bone trabeculae (B) that were mainly present in the bottom areas. Under the bone, the sinus floor was very thin, and distributed bone trabeculae were present.

Radiological examinations were performed following the principles of justification and optimization, standardizing the position of the intraoral radiographs using a silicone personalized guide [11,12].

## 4. Discussion

The phenomenon of bone resorption after dental extraction has been deeply described in several classical studies that have defined the dynamics of bone healing and the dimensional changes in the alveolus [16]. Different surgical procedures have been introduced to augment the bone and perform dental implant placements [17]. In some cases, the lateral sinus lift may be necessary if clinicians have no other options because it is an invasive procedure with risks and possible complications [18]. On the other hand, the main disadvantage of sinus elevation using the crestal approach is the uncertainty of a possible perforation of the Schneider membrane; as it is covered surgery, it is not possible to have visual control of the operation area [19].

The ideal protocol in implant dentistry is a one-stage operation to perform the implant placement immediately after the sinus lift surgery if there are clinical conditions and indications. In the present study, for personal patients’ issues, the authors agreed to adopt a delayed protocol [20,21]. This delayed implant placement allowed the authors to evaluate histologically the new bone formed during the implant site preparation [20,21]. Several techniques were introduced to improve bone healing in relation to sinus lifting surgeries by using platelet-rich plasma (PRP) [22,23,24].

However, studies about long-term implant success did not show any clinical benefits from using PRP [25,26].

Previous studies suggested using a membrane to protect the sinus from biomaterials or prevent the epithelial cells from migrating to the post-extractive alveolar site [27,28]. The actual need to use graft biomaterials to support bone formation following the lifting of the sinus membrane is highly controversial, as shown in the literature. Given the most recent scientific evidence, it is now known that graft materials are often not totally reabsorbed by the body and, consequently, not entirely replaced by newly formed bone [29]. The collagen, providing the initial structural and mechanical support to the coagulum and releasing the tension of myofibroblast in the first weeks, might prevent the premature collapse in the early stages of healing and be sufficient for alveolar socket preservation [30,31,32]. The main contractility of myofibroblasts is reported in the first 2 weeks of healing, and the resorbable propriety of collagen after this time may help in its osteoconductive action [29].

Moreover, the mechanical action of a bone fracture, during the sinus lift procedure, keeps the membrane lifted up without any artificial bone support, and instead the collagen keeps the space for the osteoinductive properties of the coagulum.

In this case report, the histomorphological analyses confirm that the quality of the new bone formation is optimal even without using graft biomaterials, while the microstructure of the bone seems to be better after the sinus lift than before. This may be due to the stabilization of the coagulum and the enlargement of the space, meaning more vascularization [33]. It is well known that the autogenous bone graft is the gold standard for new bone formation with the required quality for implant placement. Still, the extended follow-up of the study and optimal radiological results may confirm that collagen might be enough [34]. A limitation of this study is that it is not a randomized clinical trial, but only a presentation of clinical results with radiological and histomorphological data. Additionally, the small number of patients and the variable follow-up are other biases. Moreover, these patients were selected without any risk factors and with a residual maxillary bone sufficient to perform this crestal approach only using collagen sponge, but in case of larger defects, the use of biomaterials may be necessary.

In some clinical cases, less is more in regenerative dentistry, and future clinical studies are needed to confirm or to confute the histomorphological and radiological results of these cases that did not involve the use of any biomaterials.

## 5. Conclusions

With this radiological and histomorphological analyses, we have highlighted that the quality of the new healed bone is optimal and natural, with a microstructure even better than the starting bone, after 6 months from the crestal sinus lift following the use of a collagen sponge instead of bone substitutes with amino acid-based gel to manage soft tissue.

## Data Availability

Not applicable.
